# Patient-Reported Outcome Measurements in Temporomandibular Disorders and Headaches: Summary of Measurement Properties and Applicability

**DOI:** 10.3390/jcm10173823

**Published:** 2021-08-26

**Authors:** Aroldo dos Santos Aguiar, Helen Cristina Nogueira Carrer, Mariana Romano de Lira, Gabriela Zuelli Martins Silva, Thais Cristina Chaves

**Affiliations:** 1 Postgraduate Program on Rehabilitation and Functional Performance, Ribeirão Preto Medical School, University of São Paulo, Ribeirão Preto 14049-900, Brazil; aroldone@hotmail.com (A.d.S.A.); mari.rlira@hotmail.com (M.R.d.L.); zuelligabi@hotmail.com (G.Z.M.S.); 2 Department of Physical Therapy, University Federal of São Carlos, UFSCar, Rodovia Washington Luiz, Km 235-Caixa Postal 676, São Carlos 13565-905, Brazil; helencnogueira@yahoo.com.br

**Keywords:** measurement properties, temporomandibular disorders, headache, questionnaire, patient reported outcome measurements

## Abstract

Introduction: Several patient-reported outcome measurements (PROMs) are available in the literature to support the evaluation and diagnosis of temporomandibular disorders and headaches. However, clinicians and researchers usually complain that they had no education on PROMs and low overall knowledge about PROMs. Objective: This study aimed to summarize, describing the measurement properties and clinical applicability of the main condition-specific PROMs available in the literature to the assessment of patients with Temporomandibular Disorders and Headaches. Methods: The current manuscript reviewed 10 PROMs commonly used in the field. Four instruments about functioning and disability: 1. Mandibular Function Impairment Questionnaire (MFIQ), 2. Craniofacial Pain and Disability Inventory (CF-PDI), 3. 8-item and 20-item Jaw Functional Limitation Scale (JFLS), and 4. Manchester Orofacial Pain Disability Scale (MOPDS). Two instruments about headache-related disability: 5. Headache-Related Disability Index (HDI) and 6. Headache Impact Test-6 (HIT-6). Three instruments focused on TMD and headache screening: 7. 3Q/TMD, 8. Short-Form Anamnestic Fonseca Index (SFAI), 9. Headache Screening Questionnaire. In addition, one instrument about maladaptive beliefs regarding pain and injury: 10. Tampa Scale for Kinesiophobia for Temporomandibular Disorders (TSK-TMD). Conclusions: The knowledge about the limitations and applicability of the PROMs commonly used to assess TMDs and Headaches can help clinicians and researchers to obtain reliable and valid outcomes to support the decision-making process. The current review recognizes the importance of using patient-reported outcome measures in research and clinical practice. However, our findings call the attention that further studies on the measurement properties of such instruments are imperative.

## 1. Introduction

Patient-reported outcome measurements (PROMs) are recommended in the management and clinical reasoning process to guide and assess the effect of interventions and to benchmark treatment goals [1,2]. Additionally, PROMs could facilitate personalized care management, screen previously unrecognized health problems, monitor disease prognosis and disease progression, make it easier for patient–health professional communication, and promote shared decision making [3,4,5].

Several PROMs are available in the literature for the assessment of patients with Temporomandibular Disorders and Headaches. In addition, the decision to use a PROM should be supported by clinical applicability, the purpose of the instrument, and measurement properties—the degree to which an instrument measures what it is supposed to measure. [6]. The Consensus-Based Standards for the Selection of Health Measurement Instruments (COSMIN) considers three main domains to classify measurement properties: validity (the degree to which a PROM measures the construct(s) it purports to measure), reliability (the degree to which the measurement is free from measurement error), and responsiveness (the ability of a PROM to detect change over time in the construct to be measured [7].

One of the main barriers reported by clinicians that prevent the use of PROMs in clinical practice and research is the lack of training on how to use and interpret the instruments available or to judge which specific PROMs are important to use in different contexts [8]. Furthermore, clinicians usually complain that they have no education on PROMs and low overall knowledge about PROMs [8]. Considering such aspects, this study aimed to critically summarize, describing the measurement properties and clinical applicability of PROMs available in the literature to the assessment of patients with Temporomandibular Disorders and Headaches. The current manuscript reviewed 10 PROMs commonly used in the field according to purpose, content, applicability, and measurement properties:

- Four instruments about functioning and disability:
  1. Mandibular Function Impairment Questionnaire (MFIQ);
  2. Craniofacial Pain and Disability Inventory (CF-PDI);
  3. The 8-item and 20-item Jaw Functional Limitation Scale (JFLS);
  4. Manchester Orofacial Pain Disability Scale (MOPDS).
- Two instruments about headache-related disability:
  5. Headache-Related Disability Index (HDI);
  6. Headache Impact Test-6 (HIT-6).
- Two instruments focused on TMD and headache screening:
  7. Three screening questions for Temporomandibular Disorders (3Q/TMD);
  8. Short-Form Anamnestic Fonseca Index (SFAI);
  9. Headache Screening Questionnaire (HSQ).
- One instrument about maladaptive beliefs about pain and injury and movement:
  10. Tampa Scale for Kinesiophobia for Temporomandibular Disorders (TSK-TMD).

## 2. Methods and Results

This manuscript is a narrative review based on instruments for the assessment of temporomandibular disorders and headache. Four reviewers conducted the reviews, and the instruments presented here are the result of a search in the electronic databases: PubMed, Scielo, EMBASE, and Google Scholar. The criteria established for the inclusion of the PROMs in the current narrative review were:

1. The studies reporting the PROM should at least report validity and/or reliability measurements;
2. The PROM should be available and cross-culturally validated for at least two other languages other than the original language; and/or
3. The instrument should be recommended by international initiatives such as the International Network for Orofacial Pain and Related Disorders Methodology (INfORM).

The measurement properties assessed in the current study were:

- PROMs with evaluative purposes: construct validity, structural validity, reliability, internal consistency, measurement error, and responsiveness.
- PROMs with discriminative purposes: construct validity, structural validity, reliability, internal consistency, and criterion validity.

The operational definitions and the criteria to assess each measurement property adopted in the current study were based on the COSMIN criteria for good measurement properties described on the COSMIN manual for systematic reviews of PROMs and the table describing the criteria is available in the current manuscript as a Supplementary File (Table S1) [7]. Just one trained researcher applied the criteria.

We did not include several instruments in this narrative review because they did not meet our inclusion criteria. For instance, Oral Behaviors Checklist (OBC) [9] was not included in the current review because it is a checklist to assess oral behaviors that can or not be related to TMD. Moreover, the OBC was translated to just one other language.

### 2.1. PROMs to Assess Disability and Functioning

#### 2.1.1. Mandibular Function Impairment Questionnaire (MFIQ)

Purpose: The MFIQ is an instrument used to assess the patient’s perception regarding the orofacial disability [10]. It is a scale with an evaluative purpose, which means the scale aims to assess changes along the time (before and after treatment).

Content: The content of the MFIQ explores questions related to chewing, biting, and eating different foods (10 questions), yawning, kissing, drinking, laughing, speaking, and two questions about social participation (work and social activities) [10]. 

Number of items: The questionnaire has 17 structured questions [10]. 

Response options/scale: Each item is scored on a five-point ordinal scale, ranging from “no difficulty” (score = 0) to “very much difficulty or impossible without help” (score = 4) [10].

Recall period for items: No recall period is defined in the instructions of the scale. 

##### Practical Application

How to obtain: The MFIQ is fully available in the manuscript of the original publication [10]. It is in the public domain, and the tool is available free of charge. 

Method of administration: It is a self-reported instrument [10]. The original manuscript reporting the English version tested two administration methods: interview and self-administered. The authors found an acceptable correlation between the scores obtained by the two methods [10], which suggests that users can adopt both methods of administration. The MFIQ should be administered before and after treatment to compare the changes along with the time points.

Scoring: The total score is obtained by summing up the response scores of all questions as reported in the original manuscript. The instrument consists of 17 items. Although the authors divided the scale into two domains (D1: Functional Capacity and D2: Feeding), the factor analysis (structural validity) showed that the 17 items loaded on one factor [10]. As a result, we recommend using the total score obtained by summing up the score of all the 17 questions. 

Conversely, the original article describes a complex calculation method to classify the patients into masticatory function impairment subgroups. The score calculation of the MFIQ proposed by Stegenga et al. [10] considers the summing up of the score of all items of the MFIQ (simple score) and then it should be divided by the items answered by the patient to obtain the Raw Score (RS). Finally, it is used to obtain the Masticatory Function Impairment Rating. Details of the rating system can be assessed in the original publication [10]. Then the final weighted score could be used to classify in three masticatory function impairment ratings: mild impairment (0 or 1), moderate impairment (2 or 3), or severe impairment (4 or 5) (Table 1) [10]. However, it is worth noting that we could not find in the literature the validation of such classification as described in the original paper. In this way, we do not encourage the use of this scoring system.

Score interpretation: The maximum score is 68: the greater the score, the worst the masticatory system disability or function impairment. When adopting the calculation system described in the original manuscript [10], one should consider the score ranging between 0 to 5 (the greater the score, the greater the functional impairment). 

Respondent/administrative burden: We could not find any report, in the literature, regarding the time necessary for patients to fill in the instrument. 

Translations/adaptations: Beyond the English version, there are the Brazilian-Portuguese [11] and Chinese versions available in the literature [12].

##### Measurement Properties

Method of development: In the original version of the manuscript [10], it was not clearly stated how the instrument was developed (by an expert committee? Interviews with patients?). No method of concept elicitation or cognitive interviews were used to determine the content of the scale. There is a description that a preliminary version of the questionnaire was used in the clinical setting to obtain feedback from patients regarding a range of questions that were designed to assess masticatory functioning [10]. Preliminary testing of the MFIQ enrolled a sample of 95 patients with different types of TMD. The items that showed lower or higher correlations with the other instrument items were excluded from the scale. 

Reliability and internal consistency: All the versions of the MFIQ showed suitable reliability and internal consistency [11,12], except the original version [10] that did not report test-retest reliability.

Validity: The original version [10] and the Chinese version of the MFIQ [12] just assessed structural validity using exploratory factor analysis and, as a result, did not meet the criteria for suitable measurement property. 

The Brazilian-Portuguese version showed a two-factor structure (Functional Capacity = items 3, 4, 5, 8, 9, 10, and 11, and Feeding Domain = items 12–17) using confirmatory factor analysis [11] and excluding four items of the tool: 1, 2, 6, and 7. However, the model did not meet acceptable fit according to COSMIN criteria for good measurement properties (see Table 1).

The MFIQ score was not correlated with the scores of other comparator instruments —instruments that assess similar constructs (Construct Validity–Hypothesis Testing). In this way, no version met the criteria for good measurement property according to COSMIN (see Table 1).

Measurement error and responsiveness: A previous study [13] showed the smallest detectable change (SDC) of 10 units for the MFIQ (considering the context in which the MFIQ was administered on two different days). Another study calculated the minimal important change (MIC) for the MFIQ score of the Brazilian-Portuguese version and reported that a two units decrease was the minimum cutoff to detect patients who perceived a large improvement when compared with patients with no improvement on the global perceived effect scale (area under de curve (AUC) = 0.72) [14] (Table 1).

**Table 1:** Summary of the measurement properties of Mandibular Function Impairment Questionnaire (MFIQ) versions.

PROM	Authors	Study Population	Construct Validity (Hypothesis Testing)	Structural Validity	Reliability	Internal Consistency (Cronbach’s α)	Measurement Error	Responsiveness	Criterion Validity
MFIQ original	Stegenga et al. [10]	The sample consisted of patients with different types of TMD	No study found comparing MFIQ vs. comparator scales	Exploratory factor analysis—unidimensional [10]	Not reported for the original version	α = 0.95	SDC = 10 points [13]	MIC = 2 points [14]	NA
MFIQ Brazilian- Portuguese	Campos et al. [11] Calixtre et al. [13]	Patients with craniofacial complaints or diagnosed with TMD RDC/TMD	Not reported	Confirmatory factor analysis showed 2 domains: functional capacity domain (D1) and feeding domain (D2) CFI = 0.850; GFI = 0.781; RMSEA = 0.118	ICC D1: 0.89 (95% CI = 0.83–0.93) ICC D2: 0.82 (95% CI = 0.72–0.89) (test-retest interval—7 days)	α D1 = 0.87 α D2 = 0.91	Not reported	AUC = 0.72 to discriminate subgroups (patients that improved vs. patients with no change or get worse) [13]	NA
MFIQ Chinese	Xu et al. [12]	Patients with TMD according to DC/TMD	Not reported	Confirmatory factor analysis showed 3 domains RMSEA = 0.077; CFI = 0.930	ICC: 0.89 (95% CI = 0.86–0.91) (test-retest interval—7 days)	Cronbach’s α ranging from 0.9 to 0.91	Not reported	Not reported	NA
MFIQ overall quality assessment *	MFIQ Chinese and Original versions met the sufficient criterion just for 2 of 6 measurement properties MFIQ Brazilian version met the sufficient criterion just for 3 of 6 measurement properties		-	-	+	+	?	+ for Brazilian version	NA

PROM = patient-reported outcome measure, DC/TMD = Diagnostic Criteria for Temporomandibular Disorders, SDC = smallest detectable change, MIC = minimal important change, ICC = intraclass correlation coefficient, NA = not applicable. CFI = comparative fit index, TLI = Tucker Lewis index, RMSEA = root mean square error of approximation, GFI: goodness of fit index. * COSMIN quality criteria rating: “+” = sufficient, “-” = insufficient, “?” = indeterminate.

However, as the SDC reported by Kropmans et al. [13] was 10 units, we argue that 2 units decrease in the MFIQ score is not supported by statistical findings. We encourage future studies to investigate such issues further. In addition, we recommend that a decrease greater than 10 units on the MFIQ score should be considered a parameter for MIC.

Strengths/caveats and cautions/clinical and research usability: The MFIQ is a suitable tool when the clinician/researcher is particularly interested in assessing masticatory system impairment due to masticatory function-related symptoms or complaints. In this way, this instrument specifically assesses the impact of the orofacial complaints on masticatory function. If the clinician/researcher is interested in a multidimensional assessment of the impact of TMD on a patient’s life, we do not encourage the use of the MFIQ. In addition, the scale describes no recall period to guide patients on how to report their perceived limitation (last week? last month?).

The instrument could be an interesting tool for assessing disability/impairment before and after jaw and orofacial surgeries since it is focused on masticatory tasks. We need further studies on its measurement properties as it is a little bit obscure if the instrument has a one-factor structure or a two-factor structure. In addition, future studies should further check the content validity of the MFIQ and the responsiveness of the tool.

#### 2.1.2. Craniofacial Pain and Disability Inventory (CF-PDI)

Purpose: The Craniofacial Pain and Disability Inventory (CF-PDI) is a multidimensional tool that assesses at least three constructs for patients with TMD with other comorbidities: psychosocial factors, pain, and disability related to the orofacial region [15]. It is a TMD-specific tool.

Content: The original CF-PDI in Spanish is a bidimensional tool divided into two domains: the subscale Pain and Disability comprises 14 questions (1–8; 16–21), and the domain Jaw Functional Status is composed of 7 questions (9–15) [15]. The instrument also encompasses questions about headache, ear pain, and neck pain. It is a scale with an evaluative purpose, which means the scale aims to assess changes along the time (before and after treatment).

Number of items: The CF-PDI showed 21 items [15].

Response options/scale: Each question is scored on a four-point ordinal scale. The response options’ wordings vary for the different questions.

Recall period for items: No recall period is defined in the instructions of the scale.

##### Practical Application

How to obtain: The pain CF-PDI is fully available in the manuscript of the original publication. It is in the public domain, and the tool is available free of charge.

Method of administration: The CF-PDI is a self-administered tool.

Scoring: The maximum score is 63: the higher the score, the greater the TMD-related disability [15].

Score interpretation: The score is obtained by summing up the score of each question of the instrument. The CF-PDI Spanish version has two domains, and as a result, the score of each domain should be calculated separately. The domain Pain and Disability has 14 questions (1–8; 16–21), with a score ranging between 0–42, and the domain Jaw Functional Status has 7 questions (9–15), and the score ranges between 0–21.

Respondent burden: We could not find any report, in the literature, regarding the time necessary for the patients to fill in the instrument.

Translations/adaptations: The original CF-PDI is available in Spanish [15]. The instrument is also available in three other languages: Brazilian-Portuguese [16], Chinese [17], and Italian [18].

##### Measurement Properties

Method of development: In the instrument development, patients with TMD (*n* = 18) participated in a focus group and were interviewed about their perceptions of the instrument items. The draft instrument showed 30 items. After the research committee’s work, 22 items were considered in the final version of the tool covering four areas (quality of life, jaw functional status, avoidance behavior, and pain). A pilot test for cognitive debriefing was performed in 24 patients to examine the content validity of the preliminary instrument for relevance and clarity of the language. More than 96% of the patients could easily answer the questionnaire [15].

Reliability and internal consistency: All the versions of the CF-PDI showed suitable reliability and internal consistency (Table 2).

Validity: The structural validity of the original CF-PDI version was investigated by exploratory factor analysis. A two-factor solution emerged with an explained variance of 40.8%, suggesting that additional factors could better explain the construct. The domain “Pain and Disability” showed 14 questions (1–8; 16–21), and the domain “Jaw Functional Status” showed 7 questions (9–15) [15]. However, Brazilian and Italian versions met the criteria for sufficient structural validity according to COSMIN, as exploratory factor analysis is not considered in the criteria (Table 2).

The construct validity—hypothesis testing of the CF-PDI was reported on the studies of the original [15], Brazilian [16], and Italian [17] versions. However, the Brazilian [16] version described that the hypotheses raised a priori were confirmed (Table 2).

Measurement error and responsiveness: The SDC obtained for the CF-PDI Spanish version was 7 points, 11.1% of the maximal score. However, the MIC was not reported in the manuscript of the original CF-PDI [15] and the remaining version available in the literature. In this way, no version of the CF-PDI met the criteria for sufficient measurement error according to COSMIN (Table 2). We did not find any study that investigated the responsiveness of the CF-PDI (Table 2).

Strengths/caveats and cautions/clinical and research usability: The CF-PDI is a suitable instrument to assess TMD-related disability from a multidimensional perspective. It is the unique instrument available in the literature that assesses TMD patients in a multidimensional view. There is no study describing the MIC of the CF-PDI domains scores. Future studies should demonstrate the MIC values for CF-PDI. The Brazilian-Portuguese version of the CF-PDI [16] showed three domains, and one domain assesses the impact of the comorbidities on TMD patients’ life explicitly.

**Table 2:** Summary of the measurement properties Craniofacial Pain and Disability Inventory (CF-PDI) versions.

PROM	Authors	Study Population	Construct Validity (Hypothesis Testing)	Structural Validity	Reliability	Internal Consistency (Cronbach’s α)	Measurement Error	Responsiveness	Criterion Validity
CF-PDI original	La Touche et al. [15]	The study sample consisted of heterogeneous chronic craniofacial pain patients	CF-PDI total score vs. VAS: r = 0.46 CF-PDI total score vs. NDI; r = 0.65 CF-PDI vs. PCS: r = 0.46 CF-PDI vs. TSK11: r = 0.40 No hypothesis was defined	Exploratory factor analysis— 2 domains: Pain and Disability Jaw Functional Status	ICC: 0.90—(time frame between test-retest: 12 days)	α = 0.88	SDC = 7 points	No study found	NA
CF-PDI Brazilian-Portuguese	Greghi et al. [16]	A total of 100 female and male patients with temporomandibular disorders (TMD), with or without headaches	The correlations between the CF-PDI and the NDI, PCS, and TSK-TMD there were weak to moderate correlations for 94% of the comparisons (30/32). Moreover, for the correlations between the CF-PDI and the MFIQ and PDQ, there were moderate to strong correlations for 83% of the comparisons (20/24)	Confirmatory factor analysis— 3 domains: Functional and psychosocial limitation Pain Frequency of comorbidities TLI = 0.92; CFI = 0.96; RMSEA = 0.05	ICC: >0.9 (time frame between test-retest: 1 week after the initial application)	α ≥ 0.77	SDC = 5.08	No study found	NA
CF-PDI Italian	Monticome [18]	A total of 212 patients with chronic TMD	CFPDI-I strongly correlated with the NDI-I (r = 0.66, *p* < 0.05) and moderately correlated with the NPRS (r = 0.48, *p* < 0.05), PCS (r = 0.37, *p* < 0.05), TSK (r = 0.35, *p* < 0.05) and MIDAS (r = 0.47, *p* < 0.05). All hypotheses raised a priori were confirmed	Confirmatory factor analysis (CFA) showed 2 factors: Pain and Disability Jaw Functional Status GFI = 0.97; CFI = 0.98; RMSEA = 0.07	ICC: 0.86 (time frame between test-retest: after 7–10 days after the first administration)	α = 0.91	SDC = 5.5	No study found	NA
CF-PDI Chinese	Le et al. [17]	A total of 206 patients with painful temporomandibular disorders (TMD), with or without headaches	Not reported (the authors described structure validity as construct validity)	Exploratory factor analysis— 3 domains: Functional and psychosocial limitation Pain = 7 questions Frequency of comorbidities	ICC: 0.82 (time frame between test and retest: after a 4-week interval)	α = 0.91	SDC = 8.69	No study found	NA
CF-PDI overall quality assessment *	CF-PDI Brazilian and Italian versions met the sufficient criterion just for 4 of 6 measurement properties CF-PDI original and Chinese versions met the sufficient criterion just for 2 of 6 measurement properties		+ for Brazilian and Italian versions ? for the original version	+ for Brazilian and Italian versions	+	+	-	-	NA

PROM = patient-reported outcome measure, CF-PDI = Craniofacial Pain and Disability Inventory, MFIQ =Mandibular Function Impairment Questionnaire, TSK-TMD = Tampa Scale for Kinesiophobia for Temporomandibular Disorders, PCS = Pain Catastrophizing Scale, NDI = Neck Disability Index, NPRS = Numeric Pain Rating Scale, VAS = Visual Analogue Scale, MIDAS = Migraine Impact Disability Assessment, DC/TMD = Diagnostic Criteria for Temporomandibular Disorders, SDC = smallest detectable change, MIC = minimal important change, ICC = intraclass correlation coefficient, NA = not applicable, CFI = comparative fit index, TLI = Tucker Lewis index, and RMSEA = root mean square error of approximation. * COSMIN quality criteria rating: “+” = sufficient, “-” = insufficient, “?” = indeterminate.

#### 2.1.3. The 8-Item and 20-Item Jaw Functional Limitation Scale (JFLS)

Purpose: The JFLS was developed by Ohrbach et al. [19,20] to address problems identified with the existing instruments (particularly MFIQ). The instrument assesses disability related to orofacial pain, which makes it a generic tool—one can use it for different types of orofacial pain conditions. It is an instrument with an evaluative purpose (to detect change along the time). In addition, the authors argued about the difference between the constructs disability vs. functional limitation and highlighted that JFLS measures functional limitation.

Content: There are two versions of the JFLS PROM available: 8-item and 20-item JFLS [20]. The 8-item JFLS covers tasks and activities related to masticatory function, such as chewing tough and soft food, opening the mouth to drink, swallowing, yawning, talking, and smiling. The 20-item JFLS covers activities involving social aspects such as facial expressions (happy and angry), kissing, singing, frowning, laughing, and other jaw activities such as chewing a hard bread, chewing crackers, eating soft food that requires no chewing, opening the mouth wide to bite an apple or a sandwich, and talking.

Number of items: There are two versions of the JFLS instrument: one with 8 items and another with 20 items. The 8-item JFLS is the short form.

Response options/scale: The degree of limitation in carrying out activity was assessed on a numeric ordinal rating scale (0 to 10) anchored by the endpoints “no limitation” and “severe limitation” [19].

Recall period for items: The patient has been advised to answer about the scale regarding functional limitation considering the “past month” [19].

##### Practical Application

How to obtain: The 8- and 20-item JFLS in the English version are fully available accessing the link below. It is in the public domain, and the tool is available free of charge.

Method of administration: It is a self-reported scale.

Scoring: From either the short form (8-item JFLS) or the long form (20-item JFLS), a single global score of “jaw functional limitation” can be computed as the mean of the available items. The maximum score of the 8-item JFLS is 8, and the 20-item JFLS is 20. The scoring system of JFLS is described in the Scoring Manual for Self-Report Instruments—Diagnostic Criteria for Temporomandibular Disorders (DC/TMD).

Score interpretation: Higher scores denote higher jaw functional limitation.

Respondent/Administrative burden: We could not find any report, in the literature, regarding the time necessary for patients to fill in the instrument.

Translations/adaptations: The JFLS is available in English (original version), Croatian [21], and Chinese [22].

##### Measurement Properties

Method of development: The 20-item version was derived from the Buffalo Checklist, Seattle Checklist, and MFIQ. The scales were administered, and Rasch analysis was conducted to assess the relevance of the items. The 8-item was developed later. Firstly, a draft of the instrument was developed with 52 items that emerged from an expert consensus in which five physicians and researchers from the orofacial pain field participated. The questions covered subjects such as chewing, jaw function and mobility, and verbal and emotional expression. This process resulted in the 20-item JFLS. Eight patients were interviewed about the scale items’ comprehension, and the final version was tested on 132 volunteers [19].

Reliability and internal consistency: Just the Chinese version of the JFLS 20-item [19,20,21,22] met the criteria for sufficient reliability and internal consistency according to COSMIN. In addition, all the versions available met the criteria for sufficient internal consistency (Table 3).

Validity: The structural validity of the 8-item and 20-item JFLS was not adequately described in the manuscript (e.g., infit and outfit of the items). The original paper reported the Rasch analysis for the Buffalo and Seattle Checklists, similar to the final 8-item JFLS, suggesting that the scale with 8 items showed a suitable fit. The 8-item JFLS should be considered unidimensional. In addition, the definition of the dimensions of the 20-item JFLS was just based on the conceptual process rather than in measurement properties—statistical analysis; consequently, it is not possible to instruct readers properly to calculate the scores of the dimensions separately or not for the 20-item JFLS. The Chinese version of the 20-item JFLS showed three factors using confirmatory factor analysis: verbal and emotional expression (items 7, 8, 12, and 20), vertical jaw mobility (items 9 to 11 and 13 to 19), and chewing (items 1 to 6) [22]. No version of the JFLS met the criteria for sufficient measurement property according to COSMIN. The original version did not properly describe the Rasch analysis results, and the Chinese version model did not show an acceptable fit index (Table 3).

For construct validity—hypothesis testing, two versions (Original 8-item and Chinese) compared the JFLS vs. comparator PROMs [19,22]. However, both studies failed to report the hypothesis for construct validity and whether it was confirmed or not (Table 3).

Measurement error and responsiveness: No report about the SDC or MIC of the JFLS was found in the literature (Table 3).

Strengths/caveats and cautions/clinical and research usability: The JLFS strength is to cover various tasks and daily activities related to mandibular function explicitly. The recall period (past month) to report the perceived limitation increases the precision of the answers. The JFLS is a generic-type scale. The scale asks about the impairment to perform functional activities in general (“*For each of the items below, indicate the level of limitation during the past month.*”). However, patients are instructed not to report functional limitations not related to pain or difficulty. It is a scale recommended by the International Network for Orofacial Pain and Related Disorders Methodology (INfORM). The structural validity of the scale is not a consensus in the literature that prevents clear advice to clinicians and researchers on how to obtain the instrument’s score.

#### 2.1.4. Manchester Orofacial Pain Disability Scale (MOPDS)

Purpose: The MOPDS is a self-administered instrument used to assess the impact of orofacial pain on disability and social aspects, as well as several psychological symptoms. Remarkably, the questionnaire asks about the frequency that the volunteer perceives the limitation during function [23]. It is a generic PROM with an evaluative purpose.

Content: The MOPDS shows two domains: physical (7 items: 2, 3, 7, 8, 10 and 12, 13) and psychosocial (19 items: 4–6 and 17 to 26) [23]. The MOPDS covers subjects such as opening the mouth widely, allodynia, difficulty falling asleep, waking up at night, uncomfortable sleep position, difficulty eating hard food, difficulty having longer meals, no longer enjoying food, soreness to kiss, difficulty smiling, socialization problems, interruption of work, cognitive problems, problems to perform household tasks, preference to stay alone, difficult to talk for long periods, not engaging in social activities, unable to eat out in restaurants, feeling tired, embarrassed, depressed, crying easily, catastrophizing about symptoms, and difficulty in feeling pleasure in life.

Number of items: The MOPDS is composed of 26 questions.

Response options/scale: The score options range from “none of the time” (0), “on some days” (1 point), and “on most /every day (s)” (2 points).

Recall period for items: The questionnaire asks about functional and psychosocial limitations in the past month.

##### Practical Application

How to obtain: The MOPDS in the English version is fully available as an appendix in the original publication [23].

Method of administration: The MOPDS is a self-administered instrument. The Brazilian-Portuguese version was administered by an interview [24].

Scoring: The score of the questions must be summed up, and it can range from 0 to 52. As the factor analysis showed two dimensions, we recommend the use of the score of each dimension separately: physical domain (7 items: summing up the scores of the items 2, 3, 7, 8, 10 and 12, 13 = score ranges between 0 to 14) and psychosocial domain (19 items: summing up the scores of the items: 4–6 and 17 to 26 = score ranges between 0 to 38).

Score interpretation: Higher scores denote higher orofacial disability and psychosocial limitation.

Respondent/administrative burden: The manuscript of the original version reported that patients were able to complete the questionnaire in 2–3 min.

Translations/adaptations: There is the original scale in English and two other versions: Brazilian-Portuguese [24] and Arabic language [25].

##### Measurement Properties

Method of development: The MOPDS was developed by open-ended interviews with 32 patients with orofacial pain who provided a total of 100 statements that described 33 disabilities. A preliminary version with 30 statements was administered to 171 community subjects with orofacial pain and 48 patients. The final version showed 26 items.

Reliability and internal consistency: No test-retest reliability assessment was reported in the manuscript of the English version [23]. The Brazilian-Portuguese version met the criterion for sufficient test-retest reliability [24]. Both versions met the criterion for sufficient quality of the measurement property (Table 4).

Validity: For structural validity, the exploratory factor analysis retained just 26 questions since 4 questions did not show factor loadings equal to or higher than 0.4 [23]. Both the original and Brazilian versions [23,24] did not meet the criteria for sufficient quality of the structural validity (Table 4).

The paper describing the original version of the MOPDS did not report comparisons between MOPDS with other PROMs [23]. In addition, the Brazilian-Portuguese version compared the MOPDS score with the short-form oral health impact profile (OHIP-14) and with pain intensity and showed r = 0.85 and r = 0.75, respectively. No hypothesis was raised a priori, and as a result, no version met the criteria for sufficient construct validity (Table 4).

Measurement error and responsiveness: No report about the SDC or MIC of MOPDS was found in the literature (Table 4).

Strengths/caveats and cautions/clinical and research usability: MOPDS has the advantage of being a generic instrument. It is not specific to TMD patients, but it can be used to assess TMD patients [23,24]. The main disadvantage of this instrument is the nature of the response categories. It asks about frequency (the frequency in which the functional limitation is perceived) and not about the extent of perceived disability (mild limitation/severe limitation). These are different dimensions, as one patient can report a functional limitation as frequent but with mild impact in their lives. Moreover, the instrument has just three response options that may restrict the patients’ grading and make the instrument less sensitive to change.

#### 2.1.5. Headache Disability Inventory or the Henry Ford Hospital Headache Disability Inventory (HDI)

Purpose: The HDI is a multidimensional scale developed to assess the frequency of the impact of the headache on patients’ life [26]. It is a generic tool with an evaluative purpose.

Content: HDI items cover aspects related to the impact of disability on patients’ life and social impacts, and psychological issues aggravated by headaches such as fear of headache crisis or cognitive impairment [26]. The instrument reunites questions about the impact of headache on daily living activities, impact on recreational activities, the emotional impact of headaches (feeling angry, desperate, frustrated; losing control; tension; irritation), fear to engage in activities due to headache, cognitive impact, social and work impact caused by headache, difficulty in achieving goals in life, and attentional difficulties.

Number of items: The HDI has 25 questions [26].

Response options/scale: The response options are “yes” (4 points), “no” (0 points), and “sometimes” (2 points) [26].

Recall period for items: No recall period is defined in the instructions of the scale.

##### Practical Application

How to obtain: The HDI is available in the public domain with no charge: https://compassptnc.com/wp-content/uploads/2020/08/Headache.pdf (accessed on 23 July 2021).

Method of administration: The HDI is a self-administered tool, but it could be administered by an interview [26,27].

Scoring: The scoring system of the questionnaire ranges from 0 to 100 points [26].

Score interpretation: The higher the score, the greater the headache impact on routine activities as well as emotional and social aspects [26]. The total score ranges between 0 to 100 points. It is not clearly stated, but the original paper suggests two subscales for HDI: functional (11 items: 2, 4, 7, 13, 15–19, 24, and 25) and emotional (14 items: 1, 3, 5, 6, 8–12, 14, 20–23). It is implied that the use of both the total and subscales scores is accepted. However, we recommend caution when using the subscales’ score because no study has investigated the structural validity of the English version. The Brazilian-Portuguese version showed three domains: functional (items: 1, 2, 24, and 25), emotional aspects (items: 6–10, 12, 14, 16–18, 20, 22, and 23), and social participation (items: 3, 4, 11, 13, 15, 19, and 21) [27].

Respondent/administrative burden: We could not find any report, in the literature, regarding the time necessary for patients to fill in the HDI.

Translations/adaptations: The original questionnaire was developed in English, and there are versions in Spanish [28], German [29], and Brazilian-Portuguese [27]. We did not revise the measurement properties of the German version because the manuscript has been written in German.

##### Measurement Properties

Method of development: The draft version of the HDI with 40 items was derived empirically from one of the author’s clinical and research experiences. That version was administered to 108 headache patients, and the items were excluded based on Cronbach’s alpha item-total correlation. This process resulted in the 25-item HDI [26]. To exclude items considering Cronbach’s alpha item-total correlation is not a suitable procedure.

Reliability and internal consistency: The test-retest reliability of the HDI (1-week interval) [30] was calculated using a Pearson’s correlation and not the intraclass correlation coefficient as recommended. The Brazilian version [27] was the only one to meet the criterion for sufficient test-retest reliability (Table 5). On the other hand, both versions (original and Brazilian) showed acceptable internal consistency (Table 5).

Validity: Just the Brazilian version checked the structural validity of the HDI using exploratory factor analysis [27], which is not considered in the COSMIN criteria for good measurement properties (Table 5).

For construct validity—hypothesis testing, in the studies of the HDI Brazilian [27] and Spanish [28] versions, comparisons between HDI and comparator instruments were reported. However, no hypothesis was defined a priori, and as a result, no version of the HDI met the criteria for sufficient construct validity (Table 5).

Measurement error and responsiveness: In the original English version manuscript, a score for true change at a 1-week test-retest interval was 16 points at a 1-week test-retest interval [26]. The score of error was calculated based on the Bland–Altman method. According to COSMIN, no version of the HDI met the criteria for sufficient quality for measurement error, and no study describing responsiveness was found (Table 5).

Strengths/caveats and cautions/clinical and research usability: The HDI is a multidimensional instrument to assess, according to the authors, the physical and psychological limitations of headaches on a patient’s life. However, a careful look at the scale’s content makes it possible to recognize that “social participation” is also a construct of the scale. We consider that three subscales should be considered in the HDI and the Brazilian-Portuguese version confirmed such structure of the scale. It is a generic instrument and could be used for any headache type. The main limitation of the scale is the score range. The HDI has just three categories of response that may restrict its sensitivity to detect change.

#### 2.1.6. Headache Impact Test-6 (HIT-6)

Purpose: The HIT-6^TM^ questionnaire was developed by Kosinski et al. [31] to assess the headache’s impact on patients’ life. It is a generic tool with an evaluative purpose.

Content: The HIT-6^TM^ has questions covering the following issues: limitations in daily activities, needing to lie down during headaches, feeling tired, being irritated by headaches, difficulty concentrating, and the experience of pain. The questions ask about the frequency (how often) of the problems listed.

Number of items: The HIT-6 has six questions [31].

Response options/scale: The following item category weights were assigned to each HIT-6 item response: never (6 points), rarely (8 points), sometimes (10 points), very often (11 points), and always (13 points).

Recall period for items: No recall period is defined in the instructions of the scale.

##### Practical Application

How to obtain: The HIT-6™ is copyright of QualityMetric Incorporated and the GlaxoSmithKline Group of Companies. QualityMetric Incorporated performed the translations of the tool [31]. However, the English version is available online, free of charge.

Method of administration: The HIT-6^TM^ is a self-administered tool.

Scoring: The final HIT-6 score is obtained from a simple summation of the six items ranging between 36 and 78, with larger scores reflecting a more significant impact. Headache impact severity level can be categorized using score ranges based on the HIT-6 interpretation guide [32].

Score interpretation: The four headache impact severity categories are little or no impact (49 or less), some impact (50–55), substantial impact (56–58), and severe impact (60–78). However, this classification in severity categories of disability is arbitrary as no study was found in the literature supporting such classification. The manuscript describing the original version reported that a cut-point score of > 56 showed acceptable accuracy for the screening of migraine [32]. The HIT-6 correctly classified 88.8%, with sensitivity and specificity of 93.1 and 79.4%, respectively [32].

Respondent/administrative burden: As the HIT-6 is a six-item tool, it is relatively easy to complete and score. However, in the literature, we could not find any report regarding the time necessary for patients to fill in the instrument.

Translations/adaptations: There are 172 translations of the HIT-6TM, according to the QualityMetric website. However, we found manuscripts describing the process of translation and testing of measurement properties just for the following languages: Hindi [33], French [34], Persian [35], and Brazilian-Portuguese [36]. In addition, we found one manuscript reporting just the translation process of HIT-6 in 27 countries [37] and a manuscript describing that Canadian English, French, Greek, Hungarian, UK English, Hebrew, Portuguese, German, Spanish, and Dutch versions are psychometrically equivalent [38].

##### Measurement Properties

Method of development: The precursor 54 pool of items that originated the HIT was selected from the National Survey of Headache Impact (NSHI). Subsequently, the items of the HIT-6 were derived from two sources: (1) items that are most sensitive in differentiating headache impact based on an Item Response Theory (IRT) analysis conducted with existing headache disability and quality of life questionnaires, and (2) additional items that are best to characterize severe headache patients as suggested by a panel of headache clinicians [39]. Patients with headaches were interviewed by telephone (*n* = 459) and over the internet (*n* = 601) to fulfill the HIT-6 and a 41-item HIT (including the 35 items suggested by the expert clinician panel).

Reliability and internal consistency: The test-retest reliability of the HIT-6 original version was ICC = 0.78 for the total sample (*n* = 540) [31], and Cronbach’s α was 0.89 at a time one and α = 0.90 at time 2 (2 weeks apart). According to COSMIN, just the original and the Brazilian versions of the HIT-6 met the criterion for sufficient reliability (Table 6). For the internal consistency, all the versions [31,33,35,36] met the criteria for sufficient quality according to COSMIN, except for the French version [34] (Table 6).

Validity: Item Response Theory approach was adopted to derive the HIT-6 questionnaire items, and confirmatory factor analysis confirmed the unidimensional characteristic of the scale [40]. However, the confirmatory factor analysis showed an RMSEA of 0.078, which did not meet the criteria for sufficient structural validity for the HIT-6 original version (Table 8). In addition, the Rasch analysis was poorly described as the recommendations proposed by COSMIN. The Brazilian version of the HIT-6 was the only version to meet the criteria for sufficient structural validity (Table 6).

For construct validity—hypothesis testing, just the HIT-6 Persian version [35] described the expected hypothesis and confirmed them (Table 6).

Measurement error and responsiveness: The original version of the HIT-6 showed suitable measurement error and responsiveness for tension-type headaches [41]. The SDC showed values lower than MIC (SDC = 5 points and MIC = 8 points) and an AUC of 0.83 to discriminate patients who improved and not improved (Table 6). All the other versions of the HIT-6 either did not meet the criteria for suitable measurement error and responsiveness, or we cannot find studies describing such issues (Table 6).

Strengths/caveats and cautions/clinical and research usability: The HIT-6 is an easy and brief instrument to answer and complete. It is a generic instrument, but it showed a better performance in migraine patients (patients with more assumed severe headache-related disability). Although studies are reporting that it is a unidimensional instrument, in a careful analysis of its items, it is possible to find at least four different constructs (limitations in daily activities and work, emotional impact, cognitive impact, and pain severity). In addition, the questionnaire asks about the frequency of such limitations and not the extent of perceived impact. Consequently, a patient can report that a limitation often occurs, which does not necessarily mean that this limitation is highly impacting.

**Table 6:** Summary of the measurement properties of each Headache Impact Test-6-item (HIT-6) versions.

PROM	Authors	Study Population	Construct Validity (Hypothesis Testing)	Structural Validity	Reliability	Internal Consistency (Cronbach’s α)	Measurement Error	Responsiveness	Criterion Validity
HIT-6 original	Kosinski et al. [31]	The study was to test patients with headache who were members of America Online	HIT-6 vs. SF-36 scales r = −0.25 to −0.49 No hypothesis defined	Rasch analysis was used to derive the items [39] Confirmatory factor analysis RMSEA ranging from 0.078 to 0.114; two-factor Poor model fit indexes	ICC = 0.78 (Time interval= 14 days)	α = 0.89 (time 1) α = 0.90 (time 2)	For tension-type headache (TTH) SDC= 5 points MIC = 8 points [40] For migraine SDC = 9.6 points MIC = 2.5 to 6 points [41]	For tension-type headache (TTH) AUC = 0.83 to discriminate who improved vs. not improved [40]	NA
HIT-6 Hindi	Juyal et al. [33]	The sample consisted of patients with migraine	No study found	No study found	No study found	α > 0.7	No study found	No study found	No study found
HIT-6 French	Magnoux et al. [34]	The sample consisted of adult patients with chronic headaches	HIT-6 vs. MIDAS was low for both the first (0.42) and second compilations (0.44) for all patients. However, the correlation was higher for chronic headaches (0.58) compared with episodic headaches (0.48) at the first compilation and was, respectively, 0.59 and 0.42 at the second compilation No hypothesis defined	No study found	No ICC or Kappa was calculated for test-retest	No study found	No study found	No study found	No study found
HIT-6 Persian	Zandifar et al. [35]	The sample consisted of patients with episodic and chronic headaches, and they were adults	HIT-6 score in the 1st visit was negatively correlated with both mental and physical components (r = 0.39 and r = 0.35 respectively). There were significant negative correlations between SF-36 scores (mental and physical), HIT-6 total score in TTH (r = 0.31 and r = 0.34), and in migraine patients (r = 0.45 and r = 0.36). Authors anticipated that higher scores in HIT-6 would be associated with lower physical and mental component SF-36 scores and higher numbers in both headache days per month and NPRS	Not reported	No ICC or Kappa was reported for test-retest reliability	α were 0.74, 0.82, and 0.86 for the 1st, 2nd, and 3rd visits, respectively	No study found	Correlation of total HIT-6 with of NPRS between visit 1 and 2 (r = 0.18) No hypothesis defined	No study found
HIT-6 Brazilian-Portuguese	Pradela et al. [36]	The sample consisted of patients with primary and secondary headaches	HIT-6 vs. SF-12 questionnaire (r = −0.64; HIT-6 vs. frequency of headache (r = 0.22); HIT-6 vs. headache intensity (r = 0.44). No hypothesis defined	Confirmatory factor analysis revealed just one-dimension questionnaire. GFI = 0.97; CFI = 0.98; RMSEA = 0.0	ICC = 0.95	α = 0.97 with and without question number 3.	SDC individual = 4.38	NA	NA
HIT-6 overall quality assessment *	HIT-6 Original version met the criterion for 4 of 6 measurement properties HIT-6 Brazilian version met the criterion for 3 of 6 measurement properties HIT-6 Persian versions met the criterion for 2 of 6 measurement properties HIT-6 Hindi version met the criteria for 1 of 6 measurement properties		+ for the Persian version ? for original, French, Brazilian versions	+ for the Brazilian version	+ for original and Brazilian versions	+ for all versions, except for HIT-6 French version	+ for the original version for tension-type headache	+ for the original version	NA

PROM = patient-reported outcome measure, SF = Short-Form Health Survey Medical Outcomes Study, TTH = tension-type headache, MIDAS = Migraine Impact Disability Assessment, SDC = smallest detectable change, MIC = minimal important change, ICC = intraclass correlation coefficient, NA = not applicable. CFI = comparative fit index, RMSEA = root mean square error of approximation, and GFI = goodness of fit index. * COSMIN quality criteria rating: “+” = sufficient, “?” = indeterminate.

### 2.2. PROMS Used for the Screening

#### 2.2.1. Three Screening Questions for Temporomandibular Disorders (3Q/TMD)

Purpose: The 3Q/TMD was developed to help dentists detect TMD symptoms in the county of Västerbotten, Sweden. It is a questionnaire with a discriminative purpose, which means it was developed to screen patients with TMD in an easy way [42]. It is also a PROM for screening a general adult population to recognize patients needing further TMD examination and management.

Content: The 3Q/TMD has three questions as follows: Q1: “Do you have pain in your temple, face, jaw, or jaw joint once a week or more?” Q2: “Do you have pain once a week or more when you open your mouth or chew?” and Q3: “Does your jaw lock or become stuck once a week or more?”

Number of items: The questionnaire has three questions, and that explains its name (3Q/TMD).

Response options/scale: The volunteers must answer “yes” or “no” to the questions [43].

Recall period for items: The screening questions 3Q/TMD focus on weekly symptoms and signs [42].

##### Practical Application

How to obtain: The questionnaire is available in two manuscripts published previously [42,43].

Method of administration: It was not clearly stated in the manuscripts describing the validation process of the 3Q/TMD, how the instrument was administered, or any recommendation in this line. However, it is implied that it was self-administered [42].

Scoring: The scoring for each affirmative question is 1 point, then the score of the questions must be summed to obtain a score ranging between 0 to 3 [42].

Score interpretation: The positive answers for Q1 and Q2 are suggestive of painful TMD (myalgia/arthralgia) on Diagnostic Criteria for Temporomandibular Disorders (DC/TMD), and the positive answer for Q3 is suggestive of joint disorders (Disc displacements without reduction and disc displacements with reduction and intermittent locking). Individuals with an affirmative answer to at least one of the 3Q/TMD were classified as 3Q-positives. The positive answer to just one question showed an excellent negative predictive value of 0.97 (NPV = the probability that a person does not have a disease or condition, given a negative test result).

Respondent/administrative burden: We could not find any report, in the literature, regarding the time necessary for patients to fill in the instrument. However, it is a three-item questionnaire that implies it is easy to administer.

Translations/adaptations: The questionnaire was developed in Swedish idiom; however, one can find only the English version in two publications [42,43].

##### Measurement Properties

Method of development: The development of the questionnaire occurred in a two-phase study. Questions 1 and 2 were initially developed and tested in adolescents compared to Research Diagnostic Criteria for Temporomandibular Disorders (RDC/TMD) [44]. Question 3 was added to the questionnaire in an attempt to encompass joint disorders without pain.

Reliability and Internal Consistency: None study regarding the reliability of the 3Q/TMD was found in the literature (Table 7).

Validity: Two studies show the findings for the accuracy (criterion validity) of the 3Q/TMD [42,43]. The first study [42] was conducted in a general adult population. The two screening questions related to frequent pain (Q1, Q2) showed substantial validity in relation to DC/TMD pain (myalgia/arthralgia) (sensitivity = 0.52, specificity = 0.96, positive predictive value (PPV) = 0.59, and negative predictive value (NPV) = 0.95). The question related to frequent impairment of jaw function (Q3) showed fair-to-moderate validity to Temporomandibular joint (TMJ) disorders of DC/TMD (sensitivity = 0.45, specificity = 0.86, PPV = 0.15, and NPV = 0.97) [42]. The low sensitivity of the Q1 + Q2 and Q3 to detect respectively pain and joint conditions and the low PPV, particularly for the Q3, suggesting that the screening did not detect a great number of community cases living with TMD. In the second study [43], for a sample of patients referred to an Orofacial Pain Clinic, the two screening questions on pain (Q1 and Q2) were strongly associated with a pain-related TMD diagnosis (sensitivity = 0.81, specificity = 0.63, PPV = 0.69, and NPV = 0.77). For the functional screening question (Q3), the sensitivity was low, although the specificity was high (sensitivity = 0.48, specificity = 0.96, PPV = 0.92, and NPV = 0.65) [43]. In summary, in a community sample, the 3Q/TMD shows better NPV, suggesting it is suitable to discard subjects without TMD and showed good PPV when administered in the clinical setting, suggesting it is good to detect cases of TMD. The questionnaire showed reasonable accuracy (Table 7).

Measurement error and responsiveness: We cannot find studies reporting the measurement error and responsiveness of the 3Q/TMD (Table 7).

Strengths/caveats and cautions/clinical and research usability: The first strength of the 3Q/TMD is its length. Just three questions are enough to detect possible TMD cases. Secondly, it was validated compared to the DC/TMD diagnoses. Thirdly, the authors advocated that a time frame of TMD symptoms once a week or more is more clinically relevant and reliable. The screening questions 3Q/TMD focus on weekly symptoms, while the TMD pain screener from DC/TMD asks for symptoms within the last 30 days. The instrument’s limitations are the low sensitivity and PPV to detect cases in the general population and the low sensitivity of the Q3 to detect TMJ joint disorders. Studies on the reliability of the classifications of the 3Q/TMD are lacking.

#### 2.2.2. Short-Form Anamnestic Fonseca Index (SFAI)

Purpose: The Fonseca Anamnestic Index (FAI) is a PROM initially developed with 10 items in Brazilian-Portuguese [44]. It is commonly defined as a PROM to detect signs and symptoms of TMD. Therefore, FAI is a questionnaire with a discriminative purpose (detect TMD potential cases). However, the FAI showed a poor specificity performance [45]. A short-form version of the FAI (SFAI), including five questions, was tested and showed the better performance to detect myogenous TMD cases according to RDC/TMD [46] and overall TMD diagnoses according to DC/TMD [47].

Content: The SFAI has five questions as follows: Q1. “Do you have difficulty opening your mouth wide?” Q2. “Do you have difficulty moving your jaw to the sides?” Q3. “Do you feel fatigued or muscle pain when you chew?” Q4. “Do you have earaches or pain in that area (temporomandibular joint)?” and Q5. “Have you ever noticed any noise in your temporomandibular joint while chewing or opening your mouth?”

Number of items: The SFAI has five questions.

Response options/scale: The items were scored on a three-point response scale (no = 0 point, sometimes = 5 points, and yes = 10 points).

Recall period for items: No recall period for patients who answer the questionnaire is described for SFAI or FAI.

##### Practical Application

How to obtain: The questionnaire is available in Brazilian-Portuguese [48], Chinese [49], Arabic [50], Turkish [51], and Spanish [52].

Method of administration: It is assumed that it is a self-administered PROM.

Scoring: The items were scored on a three-point response scale (no = 0 point, sometimes = 5 points, and yes = 10 points). The final score is obtained by summing up the score of each question [46,47]. The score ranges between 0 to 50 points.

Score interpretation: When compared to DC/TMD, the score of 12.5 showed excellent accuracy to detect any TMD or joint TMD and 17.5 for pain-related TMD.

Respondent/administrative burden: We could not find any report, in the literature, regarding the time necessary for patients to fill in the instrument. However, it is a five-item questionnaire that implies it is easy to answer.

Translations/adaptations: The questionnaire is available in Brazilian-Portuguese [48], Chinese [49], Arabic [50], Turkish [51], and Spanish [52].

##### Measurement Properties

We just included manuscripts that checked for the measurement properties of the SFAI (short-form) since FAI (long-form) showed poor performance for screening purposes [45].

Method of development: We could not access the file describing the development of the FAI.

Reliability and internal consistency: The Chinese version [49] of the SFAI showed acceptable ICC values for Q1-Q3 and Q6, Q7 ranging from 0.51 to 0.82, and Turkish versions showed ICC values from 0.73 to 0.85. The Brazilian-Portuguese version also showed suitable reliability (ICC = 0.98) [46] and internal consistency (Cronbach’s α = 0.70) for the SFAI total score [8].

Validity: The original questionnaire demonstrated an excellent correlation between the Helkimo modified clinical index and FAI score (r = 0.95). The SFAI was obtained by structure validity analysis, showing relevant exploratory factors and Rasch analysis indexes of model fit [8]. Firstly, the SFAI accuracy in detecting TMD cases was tested against the RDC/TMD [46] and showed high accuracy to detect myogenous TMD (area under the curve of 0.97), with a better cutoff score of 17.5 points (PPV = 94.20 and PNV = 99.70). One additional study investigated the accuracy of the SFAI score to detect DC/TMD cases and reported accuracy values of 0.97 (any TMD diagnoses), 0.99 for pain-related TMD diagnoses, and 0.97 for TMD joint conditions [47]. The PPV and NPV are described in Table 8.

Measurement Error and Responsiveness: The Brazilian-Portuguese [46] version described the SDC of the total score of the SFAI (9.09) (Table 8).

Strengths/caveats and cautions/clinical and research usability: The SFAI is a short-length PROM, easy to use, and shows acceptable diagnostic accuracy compared with the RDC/TMD and DC/TMD diagnoses. However, such studies were conducted in clinical settings when the complexity of TMD cases can influence accuracy since the test’s diagnostic accuracy may be influenced by the type of population under study [53]. In this way, we recommend that further studies investigate the diagnostic accuracy validity of the SFAI score on a general population to clarify its discriminative accuracy to detect community cases. Translation and measurement properties of the FAI are described in the literature in five different languages. However, for the SFAI, just two studies [46,47] reported the measurement properties of the SFAI. Considering that the FAI score did not show acceptable diagnostic accuracy, future studies must focus on the measurement properties of the SFAI rather than the FAI. Another limitation is the absence of a recall period to patients report signs and symptoms.

#### 2.2.3. Headache Screening Questionnaire (HSQ)

Purpose: The HSQ is a questionnaire with a discriminative purpose, which means it was developed for the screening of migraine and tension-type headache (TTH) [54].

Content: The questions of the HSQ cover the following subjects: frequency of headaches, frequency of headache attacks, days with headaches, the timespan of the headache crisis, characteristic of the headache (pulsating, tight, burning, one or both sides of the head), the severity of the headaches, activities that worse the headache and avoidance of activities due to headache [54,55].

Number of items: The HSQ is a 10-item tool, showing two algorithms: to detect Migraine and TTH.

Response options/scale: Each question has different response options. The full version of the HSQ in English is fully described below.

Recall period for items: No recall period is defined in the instructions of the scale. It is implied that all life spans should be considered.

##### Practical Application

**Figure 1:**
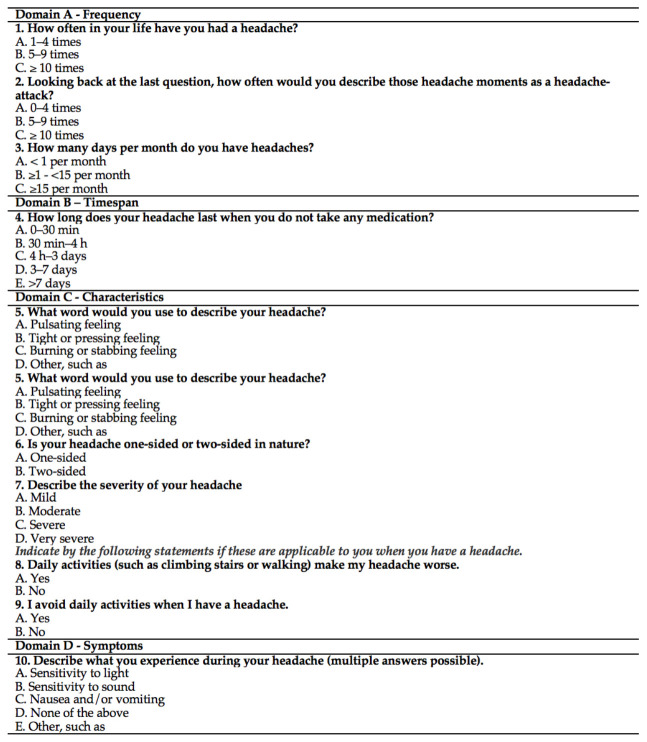
Headache Screening Questionnaire (HSQ)—English version. Reproduced from Van der Meer et al. [54].

How to obtain: The English version of the HSQ is available in the manuscripts previously published [54,55] (Figure 1).

Method of administration: The HSQ is a self-administered tool [54].

Scoring: The algorithm for Migraine and TTH diagnoses is described in Table 9.

Score interpretation: The final score is obtained by summing the scores of each question in each algorithm separately. The HSQ provides two final scores: 0–8 points for migraine and 0–8 points for TTH. If all ICHD-3 beta criteria are met for migraine and/or TTH, a person receives the maximum score of eight points for migraine and/or TTH. As people may have concurrent migraines and TTH, patients can receive eight points for each headache. When at least six points are appointed, migraine or TTH is considered “probably present”; hereafter named “probable” migraine or “probable” TTH [54].

Respondent/administrative burden: We could not find any report, in the literature, regarding the time necessary for patients to fill in the instrument. There is a burden associated with the classification algorithm use (computing the responses—see Table 9). The process to obtain the classification could be a little bit complicated.

Translations/adaptations: The English version is available in the manuscript reporting the development and validation of the instrument [54]. The Brazilian-Portuguese version is also available [56].

##### Measurement Properties

Method of development: The items were derived from the International Classification of Headache Disorders, ICHD-3 criteria [57]. Afterward, the HSQ draft version was presented to three students of physiotherapy and eight master students on orofacial physiotherapy. They tested the HSQ draft version on written case reports and each other. Finally, a cross-sectional study was conducted to test the HSQ draft version in 120 patients (55 migraines, 36 TTHs, and 29 other headaches).

Reliability and internal consistency: A study reporting the reliability and internal consistency of the instrument was not found (Table 10).

Validity: As a measure of criterion validity, the authors reported the agreement between the HSQ score vs. ICHD-3 beta diagnoses, using kappa statistics. For migraine, there was a moderate overall agreement between the ICHD-3 beta diagnoses and the HSQ (kappa = 0.58). The concomitant sensitivity was 0.69, and the specificity is 0.90. For a diagnosis of probable migraine (6 points), the overall agreement was moderate (kappa = 0.44) with a sensitivity of 0.89 and specificity of 0.54. For TTH, the overall agreement between the neurologist’s diagnosis based on the ICHD-3 beta criteria and the HSQ was fair (kappa = 0.237), the sensitivity of 0.36, and the specificity was 0.86. To detect a probable TTH (6 points), the overall agreement in the criteria was fair (kappa = 0.32). The sensitivity was 0.92, and the specificity was 0.48 [54] (Table 10).

Measurement error and responsiveness: The ability to detect change is not a dominant characteristic for screening instruments (Table 10).

Strengths/caveats and cautions/clinical and research usability: The HSQ adapted into a questionnaire the criteria ICHD-3 beta. The HSQ intends to be used in a clinical setting, such as during the physiotherapy practice. HSQ could help clinicians and other healthcare professionals screen patients with migraines and TTH and make the referral to suitable treatments. The reliability of the scores should also be investigated. The instrument needs further field testing in a bigger sample and the general population. In addition, it was found a low specificity for the TTH diagnoses.

### 2.3. PROM to Assess Beliefs and Attitudes of Fear-Avoidance

#### 2.3.1. Tampa Scale for Kinesiophobia for Temporomandibular Disorders (TSK-TMD)

Purpose: The TSK-TMD is a self-administered condition-specific instrument developed to assess maladaptive beliefs about pain, movement, and injury. The TSK-TMD is a scale with an evaluative purpose that can be used to assess patients before and after treatment.

Content: The TSK-TMD covers the following subjects: fear to move and cause jaw injury, hypervigilance, catastrophizing, movement worsening the pain, pain as a synonym of injury, worsening of symptoms and harm, fear of injury, avoidance of movement to prevent aggravating symptoms, and safety and avoidance of movement.

Number of items: The TSK-TMD is an 18-item scale. However, the confirmatory factor analysis (structural validity) showed a good fit for a 12-item structure. In this way, we recommend the administration of the 12-item scale, with two domains: Activity Avoidance (items: 1, 2, 10, 13, 15, 17, and 18) and Somatic Focus (items: 3, 5, 6, 7, and 1) [58].

Response options/scale: Each item is scored on a four-point Likert scale, ranging from “strongly disagree” (score = 1) to “strongly agree” (score = 4) [58].

Recall period for items: No recall period is defined in the instructions of the scale. It is implied that all life span should be considered.

##### Practical Application

How to obtain: The TSK-TMD is available in the public domain with no charge.

Method of administration: The TSK-TMD is a self-administered instrument.

Scoring: The score ranges between 12 to 48 points. Ratings are summed to yield a total score where the higher values reflect greater maladaptive beliefs regarding movement, pain, and injury.

Score interpretation: The higher the score obtained, the higher the maladaptive beliefs regarding movement, pain, and injury [58].

Respondent/administrative burden: We could not find any report regarding the time necessary for patients to fill in the instrument in the literature.

Translations/adaptations: There are the original versions in Dutch and English [58]. In addition, there are versions in Brazilian-Portuguese [59], Chinese [60], Korean [61], Japanese [62], and Spanish [63].

##### Measurement Properties

Method of development: Visscher et al. [58] proposed adapting the TSK original into a scale specific for TMD, considering that the general terms used in the original TSK were not suitable to meet the more localized complaints of patients with a TMD (content adaptation). The words “exercise”, “body”, and “physically active” were replaced by “jaw exercise”, “jaw”, and “using my mouth”. In addition, because TMD is a collective term embracing pain, the term “pain” from the TSK was replaced by “symptoms”. Afterward, five experts in the field of temporomandibular disorders (two dentists, two physical therapists, and a psychologist) evaluated the modified questionnaire. Finally, an independent psychologist specializing in fear of dental pain evaluated the modified version of the TSK. For reasons of clarity, some items were reformulated: “jaw exercise” was reformulated as “jaw movements”, and the words “symptoms” and “medical condition” were both reformulated as “jaw symptoms”. Ultimately, the draft version of the TSK-TMD was field-tested with 10 TMD patients (which provided no further suggestions for revision).

Reliability and internal consistency: All the versions available of the TSK-TMD [60,61,62,63] met the criteria for reliability and internal consistency, except the original version [58]. TSK-TMD original version [58] showed a Cronbach’s alpha of 0.66 for the Somatic Focus domain (lower than 0.70) (Table 11).

Validity: The structural validity of the TSK-TMD original version was assessed by confirmatory factor analysis and showed suitable model fit indexes. The best fit model showed 12 items, divided into two domains: Activity Avoidance and Somatic Focus [58]. The original [58], Brazilian [59], and Spanish [63] versions met the criteria for sufficient quality for structural validity (Table 11).

To assess hypotheses testing for construct validity, the scores on the catastrophizing pain scale were compared with the scores on the TSK-TMD original version. It was observed a positive and weak correlation between the instruments (r = 0.23) [58] (Table 12). Just the Brazilian TSK-TMD met the criteria for sufficient construct validity as the other versions failed to describe the expected hypothesis for correlations between instruments (Table 11).

Measurement error and responsiveness: It was not found in the literature any report about the MIC of TSK-TMD. However, no version of the TSK-TMD previously published met the criterion for sufficient quality of the measurement error since just SDC values were reported for the Brazilian [62] and Spanish [63] versions (Table 11). No study was found reporting the responsiveness of TSK-TMD (Table 11).

Strengths/caveats and cautions/clinical and research usability: Although the name of the scale suggests that it is an instrument to assess kinesiophobia—fear of movement—this is a misconception since no question in the scale asks about “fear of movement” or at least use the term fear. In this way, the TSK-TMD is the unique scale available in the literature to assess maladaptive beliefs about pain, movement, and injury-specific for TMD patients. It is a scale recommended by the INfORM. It is an interesting instrument to administer for evaluating the efficacy of pain education programs in which the aim is to reconceptualize maladaptive beliefs. The responsiveness of the scale score to change has not been reported in the literature yet.

**Figure 2:**
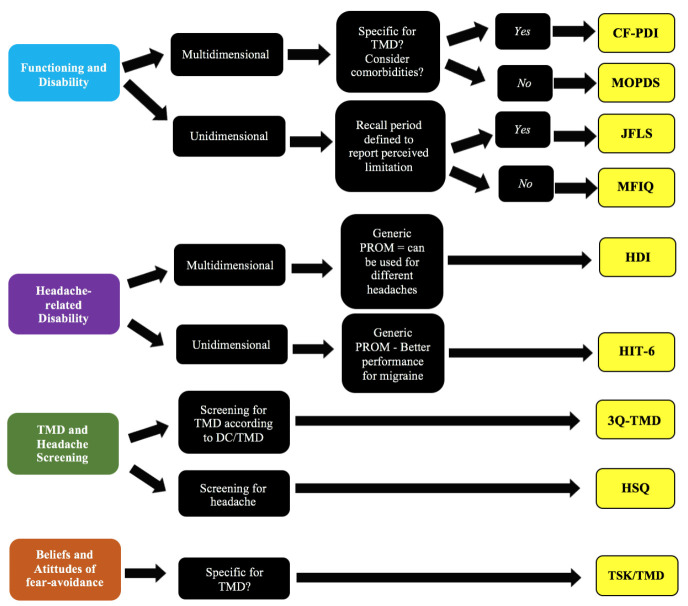
Diagram illustrating the process to choose the suitable Patient Reported Outcome Measure. Legends: PROM: Patient Reported Outcome Measure, TMD: Temporomandibular Disorders, DC/TMD: Diagnostic Criteria for Temporomandibular Disorders, MFIQ: Mandibular Function Impairment Questionnaire (MFIQ), CF-PDI: Craniofacial Pain and Disability Inventory, JFLS: Jaw Functional Limitation Scale, MOPDS: Manchester Orofacial Pain Disability Scale, HDI: Headache-Related Disability Index, HIT-6: Headache Impact Test-6, 3Q-TMD: Three screening questions for Temporomandibular Disorders (3Q/TMD), HSQ: Headache Screening Questionnaire.

In Figure 2, the reader can find a diagram with instructions on finding the best PROM to be administered in different contexts. Table 2, Table 3, Table 4, Table 5, Table 6, Table 7, Table 8, Table 9, Table 10 and Table 11 summarize the measurement properties of each PROM reviewed in the current study. In Table 12, the reader can find a brief description of the pros and cons of each PROM considered in the current review.

## 3. Discussion

Several PROMs are available in the literature to assess constructs that are significant in the context of TMD and headaches. Such instruments should be checked regarding their measurement properties. In the current review, we just choose instruments that were tested at least for validity and reliability.

The PROMs included in the current review were MFIQ, JFLS, CF-PDI, MOPDS, 3Q/TM, HSQ, HDI, HIT-6, and TSK-TMD. We included headache-related disability PROMs considering that TMD and headaches are comorbid conditions. It could be suitable to control and assess the impact of the comorbidities in patients’ lives [64] to understand the clinical picture broadly. We summarized here several instruments that may help health care professionals screen and assess outcomes during the administration of treatments.

Despite the importance of such PROMs and the constructs assessed by them, it is imperative to identify the quality of the measurement properties of such questionnaires/scales before encouraging their widespread use. A previous study [65] proposed consensus-based guidance in selecting an outcome measure in the context of a core outcome measurement set. The guidance suggests a three-step process: (1) making conceptual considerations, (2) identifying existing outcome measures, and (3) assessing the quality of the measures. To assess the quality of the measures, the interpretation of the measurement properties, e.g., reliability, validity, and responsiveness, is essential.

Keep in mind the PROMs included in the current study; our results showed a worrying scenario as most of the measurement properties of the PROMs reviewed in the current report did not meet the sufficient quality criteria described by COSMIN [7]. Particularly for the PROMs considered in the current report for assessing functioning and disability, the three versions of the MFIQ available [10,11,12] met the criteria for sufficient quality just for two measurement properties (reliability and internal consistency) of the six considered in the current report. We strongly recommend that future studies check for the construct validity properly—hypothesis testing, structural validity, measurement error, and responsiveness. For the four versions available of the CF-PDI, two versions [16,18] met the criteria for four of the six measurement properties assessed in the current review but still need to be checked regarding measurement error and responsiveness. The other two versions [15,17] met the criteria for two of the six measurements, and further studies are necessary to check for structural validity, construct validity—hypothesis testing, measurement error, and responsiveness. The JFLS 8-item and 20-item were properly checked just for internal consistency, but studies on test-retest reliability, structural validity, construct validity—hypothesis testing, measurement error, and responsiveness are still necessary. The Chinese version [22] was the unique JFLS-20 version that met the criteria for sufficient reliability. Finally, the MOPDS has just two versions available [23,24], and only internal consistency was properly checked for both. In this way, future studies should check MOPDS versions for test-retest reliability, structural validity, construct validity—hypothesis testing, measurement error, and responsiveness.

We also included two instruments to assess headache-related disability: HDI and HIT-6. The HDI versions [26,27,28] met the criteria of sufficient quality just for internal consistency, whereas the Brazilian version [27] met the criteria for reliability and internal consistency. Therefore, futures studies must assess test-retest reliability, structural validity, construct validity—hypothesis testing, measurement error, and responsiveness of the HDI. For the HIT-6, the scenario is not so cloudy. The original version of the HIT-6 met the criteria for four of six measurement properties, except for structural validity and construct validity—hypothesis testing. The Brazilian HIT-6 met the criteria for structural validity, reliability, and internal consistency, and the Persian version met the criteria just for construct validity—hypothesis testing and internal consistency. Studies on construct validity—hypothesis testing, measurement error, responsiveness, and structural validity should be carried out for the majority of the versions of the HIT-6.

For the TMD and headache screening instruments, the studies found in the literature [42,43,46,47,54] described the criterion validity adequately. One can argue that measurement properties such as internal consistency and responsiveness for PROMs with discriminative purposes (focused on signs and symptoms) are not applicable. On the other hand, we suggest that measurement error, structural validity, and reliability should be checked in future studies.

The TSK-TMD [62] Brazilian version met the criteria for the following measurement properties: construct validity—hypothesis testing, structural validity, reliability, and internal consistency. The original version [58] showed sufficient quality for structural validity and reliability, and the Spanish version [63] met the criteria for sufficient quality for structural validity and internal consistency. Measurement error, responsiveness, and construct validity—hypothesis testing still need to be checked for the majority of the TSK-TMD versions.

The CF-PDI could be highlighted for its multidimensional perspective and to cover the assessment of comorbidities related to TMD. The weakness of the MOPDS is not to assess the TMD-related comorbidities. The JFLS is the PROM recommended by INfORM to assess jaw-related disability and has the advantage of using a defined recall period to query about activity limitations. In addition, MFIQ assesses disability related explicitly related to masticatory function. As a result, CF-PDI is suitable to assess the patients considering a multidimensional perspective and TMD-related comorbidities and showed the best measurement properties. Differently from JFLS and MFIQ, which are both focused on masticatory function. In addition, MOPDS is not a TMD-specific PROM. Therefore, considering the PROMs reviewed in the current report, CF-PDI can bring a broad picture of the TMD patient.

For the headache disability assessment, both instruments are multidimensional. However, HIT-6 is shorter than HDI (which can reduce the patient burden), and it showed better performance to assess headache impact in migraine patients. Considering the widespread use of the HIT-6, its length, and its better measurement properties, we recommend the instrument preferentially for clinical and research purposes.

Screening instruments could be valuable in clinical practice. We recommend the 3Q-TMD screen for TMD because its accuracy was investigated against the DC/TMD and HSQ because it is designed based on the International Classification of Headache Disorders, ICHD-3 criteria [47]. The Fonseca Anamnestic Index (FAI) and its short-form (SFAI) are other options for screening TMD available in the literature. Notably, SFAI [47] presented high degrees of diagnostic accuracy concerning the DC/TMD to detect TMD cases. However, 3Q/TMD was tested in the community and clinical setting populations. Therefore, we need further studies to clarify the diagnostic accuracy of SFAI. In this way, in the countries in which there is a translated and validated version of the 3Q-TMD, we recommend using such PROM.

TSK-TMD is the unique instrument available in the literature to assess beliefs about pain, injury, and movement specific to TMD. The misconceptions about movement, pain, and injury should focus on the strategies to treat TMD patients. We highly recommend using TSK-TMD, particularly for patients with chronic pain TMDs (such as myalgia and arthralgia).

The current review recognizes the importance of using patient-reported outcome measures in research and clinical practice. However, our findings call the attention that further studies on the measurement properties of such instruments are imperative. Moreover, the combined administration of subjective (PROMs) and more objective measurements (such as quantitative sensory testing or performance tests) may help clinicians to minimize possible bias related to reporting such as recall bias [66] or social desirability [67].

## 4. Conclusions

In this review manuscript, we summarized the applicability and measurement properties of 10 PROMs designed with evaluative and discriminative purposes for patients with Temporomandibular Disorders and Headaches. The current review recognizes the importance of using patient-reported outcome measures in research and clinical practice. However, our findings call the attention that further studies on the measurement properties of such instruments are imperative.

## Figures and Tables

**Table 3:** Summary of the measurement properties of each Jaw Functional Limitation Scale (JFLS) version.

PROM	Authors	Study Population	Construct Validity (Hypothesis Testing)	Structural Validity	Reliability	Internal Consistency (Cronbach’s α)	Measurement Error	Responsiveness	Criterion Validity
JFLS-8 original	Ohrbach et al. [19]	The study was tested in individuals with TMD of university clinics	JFLS-8 vs. SCL-90 somatization: r = 0.33 JFLS-8 vs. characteristic pain GCPS: r = 0.51 JFLS-8 vs. pain interference GCPS: r = 0.52 JFLS-8 vs. Jaw symptom index: r = 0.62 No hypothesis defined	Rasch analysis—unidimensional [19]—did not describe all the items recommended by COSMIN criteria for good measurement properties Did not meet all criteria for suitable Rasch analysis	CCC = 0.81 [19] (time interval = 1 to 2 weeks) Statistic is not suitable	α = 0.87 [19]	No study found	No study found	No study found
JFLS-20 original	Ohrbach et al. [20]	The sample consisted of patients with different types of diagnostic (TMD, primary Sjögren syndrome, burning mouth syndrome, skeletal malocclusion, and healthy controls)	No study found comparing JFLS vs. comparator scales	Not accurately described for the original version	CCC = 0.87 Statistic is not suitable	α = 0.95	No study found	No study found	NA
JFLS-20 Croatian	Fetai et al., 2020 [21]	The sample comprised patients with TMD and non-patients	Not reported	Exploratory factor analysis showed 3 domains: chewing, mobility, and communication	Not reported	α calculated for items (not for domain’s score) ranging from 0.81 to 0.93	Not reported	Not reported	NA
JFLS-20 Chinese	Xu et al., 2020 [22]	Patients with TMD diagnosed according to DC/TMD	JFLS vs. MFIQ: 0.809 JFLS vs. PHQ—9: 0.405 JFLS vs. PHQ—15: 0.379 JFLS vs. GAD—7: 0.358 No hypothesis defined	Confirmatory factor analysis showed 3 domains: facial expression—DI, vertical mobility—D2, and chewing—D3 RMSEA = 0.091, CFI = 0.896	ICC D1: 0.85 ICC D2: 0.88 ICC D3: 0.89	α = 0.9	Not reported	Not reported	NA
JFLS-8 overall quality assessment	JFLS-8 version met the sufficient criterion just for 2 of 6 measurement properties		?	?	-	+	-	-	?
JFLS-20 overall quality assessment *	JFLS-20 original and Croatian versions met the sufficient criterion just for 1 of 6 measurement properties JFLS-20 Chinese version met the sufficient criterion for 2 of 6 measurement properties		?	?	+ for the Chinese version	+	?	?	NA

PROM = patient-reported outcome measure, MFIQ = Mandibular Function Impairment Questionnaire, TSK-TMD = Tampa Scale for Kinesiophobia for Temporomandibular Disorders, PCS = Pain Catastrophizing Scale, NDI = Neck Disability Index, NPRS = Numeric Pain Rating Scale, VAS = Visual Analogue Scale, MIDAS = Migraine Impact Disability Assessment, GCPS = Graded Chronic Pain Scale, PHQ = Patient Health Questionnaire, GAD = generalized anxiety disorder, CFI = comparative fit index, RMSEA: root mean square error of approximation, DC/TMD = Diagnostic Criteria for Temporomandibular Disorders, SDC = smallest detectable change, MIC = minimal important change, ICC = intraclass correlation coefficient, and NA = not applicable. * COSMIN quality criteria rating: “+” = sufficient, “-” = insufficient, “?” = indeterminate.

**Table 4:** Summary of the measurement properties of the Manchester Orofacial Pain Disability Scale (MOPDS) versions.

PROM	Authors	Study Population	Construct Validity (Hypothesis Testing)	Structural Validity	Reliability	Internal Consistency (Cronbach’s α)	Measurement Error	Responsiveness	Criterion Validity
MOPDS original	Aggarwal et al. [23]	The study was tested on community subjects with self-reported orofacial pain and dental hospital patients	Original version = no reported comparisons between MOPDS vs. comparator scales	Exploratory factor analysis— 2 domains: Physical (7 items) Psychosocial (19 items)	Not reported for the original version	Physical domain: α = 0.78 Psychosocial domain: α = 0.92	No study found	No study found	NA
MOPDS Brazilian	Kallás et al. [24]	The study sample included 50 patients with orofacial pain	MOPDS vs. OHIP-14: r = 0.857 MOPDS vs. VAS: r = 0.758	Not reported	ICC = 0.924 (Time interval = 15–20-day interval)	(Cronbach’s α = 0.9), inter-observer (ICC = 0.92) and intra-observer (ICC = 0.98)	NA	No study found	NA
MOPDS overall quality assessment *	MOPDS Brazilian version met the sufficient criteria for 2 of 6 measurement properties MOPDS original version met the sufficient criteria for 1 of 6 measurement properties		? just for MOPDS Brazilian version	?	+ just for MOPDS Brazilian version	+	?	?	NA

PROM = patient-reported outcome measure, OHIP = Oral Health Impact Profile, VAS = Visual Analogue Scale, SDC = smallest detectable change, MIC = minimal important change, ICC = intraclass correlation coefficient, and NA = not applicable. * COSMIN quality criteria rating: “+” = sufficient, “?” = indeterminate.

**Table 5:** Summary of the measurement properties of Headache Disability Inventory (HDI) versions.

PROM	Authors	Study Population	Construct Validity (Hypothesis Testing)	Structural Validity	Reliability	Internal Consistency (Cronbach’s α)	Measurement Error	Responsiveness	Criterion Validity
HDI original	Jacobson et al. [26]	The study tested headache patients	It was reported a significant main effect between headache severity vs. HDI total score (F = 8.52, df = 2, *p* < 0.001) Original version = no reported comparisons between HDI vs. comparator scales No hypothesis defined	No study found	r = 0.76 (1-week time interval) r = 0.83 (60-day time interval)	α = 0.94 [26]	Error: 16 points (Bland–Altman method)	No study found	NA
HDI Brazil	Pradela et al. [27]	The sample consisted of patients with primary and secondary headaches diagnosed by neurologists, and the patients were between 18 and 65 years old	HDI vs. SF-12 questionnaire (r = −0.70; HDI vs. HIT-6 questionnaire (r = 0.67); HDI vs. frequency of headache (r = 0.07). No hypothesis defined	Exploratory factor analysis— 3 domains: emotional = 14 questions; functional = 4 questions; and social = 7 questions	ICC = 0.95 interval from 1–3 weeks between the first and second applications	Total α = 0.84; functional domain α = 0.86 and emotional domain α = 0.88	SDC was not reported	No study found	NA
HDI Spanish	Franco et al., 2000 [28]	The sample consisted of adult patients with primary headaches	Correlation between the two domains of the HDI Spanish (r = 0.73), the correlation between the domains of the HDI Spanish vs. The Pain Behavior Questionnaire Spanish version (r = 0.38 to 0.57) No hypothesis defined	Principal component Factor Analysis revealed two factors for the HDI Spanish: functional domain (14 items) and emotional domain (11 items)	No study found	total α = 0.94; functional and emotional domains α = 0.91	No study found	No study found	No study found
HDI overall quality assessment *	HDI versions met the sufficient criterion just for 1 of 6 measurement properties Brazilian version met the criteria for 2 of 6 measurement properties		? for Brazilian and Spanish versions	?	+ for the Brazilian version	+	?	?	NA

PROM = patient-reported outcome measure, HIT-6 = Headache Impact Test, SDC = smallest detectable change, MIC = minimal important change, ICC = intraclass correlation coefficient, and NA = not applicable. * COSMIN quality criteria rating: “+” = sufficient, “?” = indeterminate.

**Table 7:** Summary of the measurement properties of 3Q/TMD: Three screening questions for Temporomandibular Disorders (3Q/TMD) versions.

PROM	Authors	Study Population	Construct Validity (Hypothesis Testing)	Structural Validity	Reliability	Internal Consistency (Cronbach’s α)	Measurement Error	Responsiveness	Criterion Validity
3Q/TMD original	Lövgren et al. [42,43]	The first study was conducted in a general adult population. In the second study, for a sample of patients referred to an orofacial pain clinic.	No study found	No study found	No study found	NA	No study found	No study found	General adult population [42]: **For pain TMD diagnoses (Q1 + Q2)** Sensitivity = 0.52 Specificity = 0.96 PPV = 0.59, NPV = 0.95 **For TMJ joint disorders (Q3)** Sensitivity = 0.45, specificity = 0.86 PPV = 0.15, NPV = 0.97 Clinical setting [43]: **For pain TMD diagnoses (Q1 + Q2)** Sensitivity = 0.81, specificity = 0.63 PPV = 0.69, NPV = 0.77 **For TMJ joint disorders (Q3)** Sensitivity = 0.48, specificity = 0.96 PPV = 0.92, NPV = 0.65
3Q/TMD overall quality assessment *	3Q/TMD met the criterion for 1 of 6 measurement properties that can be checked for discriminative purpose instruments **		?	?	?	NA	?	?	+

PROM = patient-reported outcome measure, PPV = positive predictive value, NPV = negative predictive value, TMD = temporomandibular disorders, TMJ: temporomandibular joint, TTH = tension-type headache, and NA = not applicable. * COSMIN quality criteria rating: “+” = sufficient, “?” = indeterminate. ** For several PROMs designed with discriminative purposes, one can argue that several measurement properties are not suitable such as internal consistency or construct validity or responsiveness.

**Table 8:** Summary of the measurement properties of Short-Form Fonseca Anamnestic Index (SFAI).

PROM	Authors	Study Population	Construct Validity (Hypothesis Testing)	Structural Validity	Reliability	Internal Consistency (Cronbach’s α)	Measurement Error	Responsiveness	Criterion Validity
FAI—short-form Brazilian-Portuguese	Pires et al. [46]	Myogenous TMD according to RDC/TMD	No study found	Exploratory factor analysis showed three dimensions and resulted in the 5-item short form (SFAI). In addition, Rasch analysis showed good fit for the SFAI	ICC: 0.98 (time (interval for retest—7 days)	NA	SEM = 3.28	MDC = 9.09	**DTM Miogênica (AUC = 0.97, 95%CI = 0.95–0.99)** Standard error = 0.02 Cutoff point = 17.05 Sensitivity = 0.86 Specificity = 0.95 PPV = 0.94 NPV = 0.99
FAI—short-form Chinese	Yap et al. [47]	TMD patients diagnosed according to DC/TMD (*n* = 866 and TMD-free *n* = 57)	No study found	No study found	No study found	NA	No study found	No study found	**All TMDs (AUC = 0.97)** Cutoff Point = 12.5 sensitivity = 0.91 specificity = 0.93 PPV = 0.99, NPV = 0.41 **Pain-related TMDs (AUC = 0.99)** Cutoff Point = 17.5 sensitivity = 0.97 specificity = 0.96 PPV = 0.99, NPV = 0.83 **Intra-articular TMDs (AUC = 0.97)** Cutoff Point = 12.5 sensitivity = 0.90 specificity = 0.93 PPV = 0.99, NPV = 0.42

PROM = patient-reported outcome measure, PPV = positive predictive value, NPV: negative predictive value, TMD = temporomandibular disorders, TMJ = temporomandibular joint, NA = not applicable, and MDC = minimum detectable change.

**Table 9:** The Headache Screening Questionnaire Algorithm for Migraine and Tension-type Headache (TTH).

	Algorithm for Migraine	Algorithm for TTH
Question 1	Not applicable	Alternative C (2 points)
Question 2	Alternatives B (2 points) or C (2 points)	Not applicable
Question 4	Alternative C (2 points)	Alternatives B to E (2 points)
Question 5	Alternative A (1 point)	Alternative B (1 point)
Question 6	Alternative A (1 point)	Alternative B (1 point)
Question 7	Alternatives B (1 point) or C (1 point)	Alternative B (1 point)
Question 8	Alternative A (1 point)	Alternative B (1 point)
Question 9	Alternative A (1 point)	Not applicable
Question 10	Alternatives B (1 point) or C (2 points)	Alternatives A and B (1 point) and alternatives D and E (2 points)

Question 3 is used just to determine the chronicity of headaches.

**Table 10:** Summary of the measurement properties of each HSQ and its versions.

PROM	Authors	Study Population	Construct Validity (Hypothesis Testing)	Structural Validity	Reliability	Internal Consistency (Cronbach’s α)	Measurement Error	Responsiveness	Criterion Validity
HSQ—original	van der Meer et al. [54]	Patients with headaches and non-patients were enrolled	No study found	No study found	No study found	NA	No study found	No study found	**For migraine:** Sensitivity = 0.69 Specificity= 0.90 **For probable migraine:** Sensitivity = 0.89 Specificity = 0.54 **For TTH:** Sensitivity = 0.36 Specificity = 0.86 **For probable TTH:** Sensitivity = 0.92 Specificity = 0.48
HSQ—Brazilian-Portuguese	Lopes et al. [57]	The sample consisted of patients with and without headache and over 18 years old	No study found	No study found	Kappa = 0.80 for tension-type headache diagnoses and 0.88 for migraine. (Time interval = 7 days)	NA	No study found	No study found	No study found
HSQ—English	van der Meer et al. [54]	No study found	No study found	No study found	No study found	NA	No study found	No study found	No study found
HSQ overall quality assessment *	HSQ original and Brazilian versions met the criterion for 1 of 6 measurement properties that can be checked for discriminative purpose instruments **		?	?	+ for HSQ—Brazilian	NA	?	?	+ for HSQ original

PROM = patient-reported outcome measure, PPV = positive predictive value, NPV = negative predictive value, TTH = tension-type headache, NA = not applicable. * COSMIN quality criteria rating: “+” = sufficient, “?” = indeterminate. ** For several PROMs designed with discriminative purpose one can argue that several measurement properties are not suitable such as internal consistency or construct validity or responsiveness.

**Table 11:** Summary of the measurement properties of Tampa Scale for Kinesiophobia for Temporomandibular Disorders (TSK-TMD) versions.

PROM	Authors	Study Population	Construct Validity (Hypothesis Testing)	Structural Validity	Reliability	Internal Consistency (Cronbach’s α)	Measurement Error	Responsiveness	Criterion Validity
TSK-TMD original	Visscher et al. [58]	The sample consisted of patients with TMD	TSK-TMD vs. PCS: r = 0.23 No hypothesis defined	Confirmatory factor analysis showed [47]: 2 domains—12 items, 2 domains: Activity Avoidance and Somatic Focus CFI = 0.95; RMSEA = 0.078;	ICC total score = 0.73 ICC Activity Avoidance score = 0.67 ICC Somatic Focus score = 0.71 (Time interval = 4 weeks)	Activity Avoidance domain α = 0.82 Somatic Focus domain α = 0.66	Not reported	No study found	NA
TSK-TMD-Brazilian-Portuguese	Aguiar et al. [59]	100 female patients with chronic TMD	TSK-TMD vs. PCS: r = 0.48 TSK-TMD vs. PHQ-8: r = 0.38 TSK-TMD vs. MFIQ: r = 0.43 84% of the hypotheses were confirmed	Confirmatory factor analysis showed: 2 domains—12 items, two domains: Activity Avoidance Domain and Somatic Focus GFI = 0.94; CFI = 0.97; RMSEA = 0.07	ICC total score = 0.95 ICC Activity Avoidance score = 0.93 ICC Somatic Focus score = 0.95 (Time interval = 1 week)	Activity Avoidance domain α = 0.78 Somatic Focus domains α = 0.78	Activity Avoidance score SDC = 2.78 Somatic Focus score SDC = 2.55	No study found	NA
TSK-TMD-Chinese	He et al. [60]	A total of 160 patients with TMD	Significant correlations were observed between TSK-TMD scores and global oral health rating (*r* = 0.46–0.55) No hypothesis defined	Exploratory factor analysis confirmed the bidimensional structure: Activity Avoidance domain and Somatic Focus domain	ICC total score = 0.797 ICC activity avoidance score = 0.807 ICC somatic focus score = 0.760 (Time interval = 2 weeks)	Cronbach’s alpha for the whole score of TSK-TMD was 0.919 and values of the subscales ranged from 0.895 for somatic focus to 0.907 for activity avoidance	NA	No study found	NA
TSK-TMD-Korean	Park et al. [61]	A total 90 subjects (50 women, 40 men)	Not reported	Not reported	ICC total score = 0.752 ICC activity avoidance score = 0.722 ICC somatic focus score = 0.677 (Time interval = 1/2 weeks)	Score total α = 0.858 Activity Avoidance domain α = 0.838 Somatic Focus domains α = 0.807	NA	No study found	NA
TSK-TMD-Japanese	Uritani et al. [62]	101 patients with TMD (84 women and 17 men) The mean treatment period was 22.1 months	Not reported	Not reported	ICC was 0.82	Cronbach’s alpha of the questionnaire was more than 0.7	NA	No study found	NA
TSK-TMD-Spanish	La Touche et al. [63]	The study sample included 110 patients with TMD	TSK-TMD vs. CF-PDI: r = 0.511 TSK-TMD vs. PCS: r = 0.413 TSK-TMD vs. VAS: r = 0.335 TSK-TMD vs. TSK-11: r = 0.563 TSK-TMD vs. Maximum Mouth opening: r = −0.406 No hypothesis defined	Confirmatory factor analysis—CFI = 0.989; TLI= 0.986; RMSEA = 0.078 2 domains (10 items): Activity Avoidance and Somatic Focus	ICC total score = 0.843 ICC Activity Avoidance score = 0.938 ICC Somatic Focus score = 0.885 (Time interval = 10 days later)	Score total α = 0.843 Activity Avoidance domain α = 0.938 Somatic Focus domains α = 0.885	SDC95 total score = 6.25 Activity Avoidance score SDC = 5.09 Somatic Focus score SDC = 5.57		NA
TSK-TMD overall quality assessment *	TSK-TMD Brazilian version met the criterion for 4 of 6 measurement properties TSK-TMD original and Spanish versions met the criterion for 2 of 6 measurement properties		+ just for the Brazilian version ? original, Chinese and Spanish versions	+ for TSK-TMD original, Brazilian and Spanish versions	+	+ for all versions, except the original TSK-TMD	?	-	NA

PROM = patient-reported outcome measure, MFIQ = Mandibular Function Impairment Questionnaire, PCS = Pain Catastrophizing Scale, GCPS = Graded Chronic Pain Scale, TMD = temporomandibular disorders, TMJ = temporomandibular joint, NPRS = Numeric Pain Rating Scale, VAS = Visual Analogue Scale, CFI = comparative fit index, TLI = Tucker Lewis index, RMSEA = root mean square error of approximation, GFI: goodness of fit index, SDC = smallest detectable change, MIC = minimal important change, ICC = intraclass correlation coefficient, and NA = not applicable. * COSMIN quality criteria rating: “+” = sufficient, “-” = insufficient, “?” = indeterminate.

**Table 12:** Pros and cons of the patient-reported outcome measures included in the current review.

	Pros	Cons
TMD Functioning and Disability		
Mandibular Function Impairment Questionnaire (MFIQ)	Specific to masticatory-related disability and functioning It is short and easy to use	Does not consider the impact of TMD on social or emotional aspects No recall time for report Responsiveness has not been checked The structure of the PROM is not clear (Should I use the total score or the domain scores?)
Craniofacial Pain and Disability Inventory (CF-PDI)	It is multidimensional PROM, encompassing not just masticatory function impairment but social, psychological, and the impact of comorbidities in TMD patients’ life	Responsiveness has not been checked No recall time for report
8-item and 20-item Jaw Functional Limitation Scale (JFLS)	Specific to masticatory-related disability and functioning Recall period (past month) to report the perceived limitation increases the precision of the answers It is a scale recommended by the INfORM	The structure of the PROM is not clear (Should I use the total score or the domain scores?)
Manchester Orofacial Pain Disability Scale (MOPDS)	It is a generic PROM, not specific to TMD	The response categories do not ask the extent/degree of the perceived disability Just three response categories—less sensitive to capture changes
**Headache-related disability**		
Headache-Related Disability Index (HDI)	It is a multidimensional PROM, including the psychosocial impact of headaches It is a generic PROM, not specific to one headache type	The structure of the PROM is not clear (Should I use the total score or the domain scores?) Just three response categories—less sensitive to capture changes
Headache Impact Test-6 (HIT-6)	It is short and easy to use It is a generic PROM, not specific to one headache type	Different constructs without considering different domains in the scale The response categories do not ask the extent/degree of the perceived disability
**TMD and headache screening**		
Three screening questions for Temporomandibular Disorders (3Q/TMD)	It is short and easy to use and can improve the screening It was validated compared to the DC/TMD diagnoses The time frame focused on weekly symptoms could be more clinically relevant and reliable than the “pain in the last 30 days” Its diagnostic accuracy validity was checked in the general population and in the clinical setting	Low sensitivity and PPV to detect cases in the general population and low sensitivity of question 3 (Q3) to detect TMJ disorders No study on the reliability of the diagnoses
Short-Form Anamnestic Fonseca Index (SFAI)	It is short and easy to use and can improve the screening It was validated compared to the DC/TMD diagnoses It was available in 5 different languages	Its diagnostic accuracy validity was checked just in the clinical setting No recall time for report The majority of the studies reported measurement properties for the long version (FAI) and not for the short-form (SFAI). FAI score did not show good diagnostic accuracy
Headache Screening Questionnaire (HSQ)	Based on the ICHD-3 beta It is short and easy to use and can increase the screening strategies	No study on the reliability of the diagnoses The instrument needs further field testing in a bigger sample and in the general population Low specificity for the TTH diagnoses
**Beliefs about pain, injury, and movement**		
Tampa Scale for Kinesiophobia for Temporomandibular Disorders (TSK-TMD)	It is specific to TMD The TSK-TMD is the unique scale available in the literature to assess maladaptive beliefs about pain It is a scale recommended by the INfORM The scale can be used to assess pain reconceptualization after pain education programs	The name of the scale suggests it measures kinesiophobia—but it assesses beliefs about pain and movement. There is no question in the scale that asks about “fear of movement” or at least use the term fear Responsiveness has not been checked

PROM = patient-reported outcome measure, TMD = temporomandibular disorders, TTH = tension-type headache, ICHD-3 = International Classification for Headache Disorders, DC/TMD = Diagnostic Criteria for TMD, and INfORM = International Network for Orofacial Pain and Related Disorders Methodology.

## Data Availability

No new data were created or analyzed in this study. Data sharing is not applicable to this article.

## Supplementary Materials

The following are available online at https://www.mdpi.com/article/10.3390/jcm10173823/s1, Table S1: Quality criteria for good measurement properties according to COSMIN manual for systematic reviews.
