# Supplementary material for: Patient-Reported Outcome Measurements in Temporomandibular Disorders and Headaches: Summary of Measurement Properties and Applicability
Source: J Clin Med. 2021 Aug 26;10(17):3823. doi: 10.3390/jcm10173823 (PMC8432093; doi:10.3390/jcm10173823)
Supplement: Supplementary file 1 [file jcm-10-03823-s001.zip › jcm-1347724-supplementary.pdf]

Table S1. Quality criteria for good measurement properties according to COSMIN manual for systematic reviews. Reproduced from Prinsen et al. [7].

Measurement Property	Rating	Criteria
Construct Validity (hypothesis testing)	+	The result is in accordance with the hypothesis
	?	No hypothesis defined (by the review team)
	-	The result is not in accordance with the hypothesis
Structural Validity	+	**CTT:**<br>CFA: CFI or TLI or comparable measure >0.95 OR RMSEA <0.06 OR SRMR <0.082<br>**IRT/Rasch:**<br>No violation of unidimensionality: CFI or TLI or comparable measure >0.95 OR RMSEA <0.06 OR SRMR <0.08<br>AND<br>no violation of local independence: residual correlations among the items after controlling for the dominant factor < 0.20 OR Q3's <0.37<br>AND<br>no violation of monotonicity: adequate looking graphs OR item scalability >0.30<br>AND<br>adequate model fit:<br>IRT: $\chi 2$ >0.01<br>Rasch: infit and outfit mean squares ≥ 0.5 and ≤ 1.5 OR Z standardized values > -2 and <2
	?	CTT: Not all information for '+' reported<br>IRT/Rasch: Model fit not reported
	-	Criteria for '+' not met
Reliability	+	ICC or weighted Kappa ≥ 0.70
	?	ICC or weighted Kappa not reported
	-	ICC or weighted Kappa < 0.70
Internal Consistency*	+	At least low evidence for sufficient structural validity AND Cronbach's alpha(s) ≥ 0.70 for each unidimensional scale or subscale
	?	Criteria for "At least low evidence for sufficient structural validity" not met
	-	At least low evidence for sufficient structural validity AND Cronbach's alpha(s) < 0.70 for each unidimensional scale or Subscale
Measurement Error	+	SDC or LoA < MIC
	?	MIC not defined
	-	SDC or LoA > MIC
Responsiveness	+	The result is in accordance with the hypothesis OR AUC ≥ 0.70
	?	No hypothesis defined (by the review team)
	-	The result is not in accordance with the hypothesis7 OR AUC <0.70
Criterion Validity**	+	Correlation with gold standard ≥ 0.70 OR AUC ≥ 0.70
	?	Not all information for '+' reported
	-	Correlation with gold standard < 0.70 OR AUC < 0.70

* We did not consider the "structural validity criterion" necessary to rate internal consistency
** We considered the description of sensitivity and specificity as suitable for criterion validity of PROM with discriminative purpose. CTT: Classical Test Theory, IRT: Item Response Theory, ICC:

Intraclass Correlation Coefficient, CFI: Comparative fit index, TLI: Tucker Lewis index and RMSEA: Root Mean Square Error of Approximation, SDC: Smallest Detectable Change, MIC: Minimal Important Change, AUC: Area Under the Curve, LoA: Limits of Agreement, SRMR: Standardized Root Mean Squared Residual. Rating: "**+** " = sufficient, "**-**"= insufficient, "**?**" = indeterminate
